# Diagnostic and prognostic value of plasma neurofilament light and total-tau in sporadic Creutzfeldt-Jakob disease

**DOI:** 10.1186/s13195-021-00815-6

**Published:** 2021-04-21

**Authors:** Inga Zerr, Anna Villar-Piqué, Peter Hermann, Matthias Schmitz, Daniela Varges, Isidre Ferrer, Joachim Riggert, Henrik Zetterberg, Kaj Blennow, Franc Llorens

**Affiliations:** 1 Department of Neurology, National Reference Center for TSE Surveillance, University Medical Center, Robert-Koch Street 40, Göttingen, Germany; 2 German Center for Neurodegenerative Diseases (DZNE), Göttingen, Germany; 3 Center for Networked Biomedical Research in Neurodegenerative Diseases (CIBERNED), L’Hospitalet de Llobregat, Feixa Llarga s/n, Barcelona, Spain; 4 Bellvitge Biomedical Research Institute (IDIBELL), L’Hospitalet de Llobregat, Spain; 5 Department of Pathology and Experimental Therapeutics, University of Barcelona, L’Hospitalet de Llobregat, Spain; 6 Department of Transfusion Medicine, University Medical School, Göttingen, Germany; 7 Department of Psychiatry and Neurochemistry, Institute of Neuroscience and Physiology, The Sahlgrenska Academy at the University of Gothenburg, Mölndal, Sweden; 8 Clinical Neurochemistry Laboratory, Sahlgrenska University Hospital, Mölndal, Sweden; 9 Department of Neurodegenerative Disease, UCL Institute of Neurology, London, UK; 10 UK Dementia Research Institute, London, UK

**Keywords:** Dementia, Creutzfeldt-Jakob disease, Biomarkers, Plasma, Neurofilament light, Tau, Diagnosis, Disease progression

## Abstract

**Background:**

Blood neurofilament light (Nfl) and total-tau (t-tau) have been described to be increased in several neurological conditions, including prion diseases and other neurodegenerative dementias. Here, we aim to determine the accuracy of plasma Nfl and t-tau in the differential diagnosis of neurodegenerative dementias and their potential value as prognostic markers of disease severity.

**Methods:**

Plasma Nfl and t-tau were measured in healthy controls (HC, *n* = 70), non-neurodegenerative neurological disease with (NND-Dem, *n* = 17) and without dementia syndrome (NND, *n* = 26), Alzheimer’s disease (AD, *n* = 44), Creutzfeldt-Jakob disease (CJD, *n* = 83), dementia with Lewy bodies/Parkinson’s disease with dementia (DLB/PDD, *n* = 35), frontotemporal dementia (FTD, *n* = 12), and vascular dementia (VaD, *n* = 22). Biomarker diagnostic accuracies and cutoff points for the diagnosis of CJD were calculated, and associations between Nfl and t-tau concentrations with other fluid biomarkers, demographic, genetic, and clinical data in CJD cases were assessed. Additionally, the value of Nfl and t-tau predicting disease survival in CJD was evaluated.

**Results:**

Among diagnostic groups, highest plasma Nfl and t-tau concentrations were detected in CJD (fold changes of 38 and 18, respectively, compared to HC). Elevated t-tau was able to differentiate CJD from all other groups, whereas elevated Nfl concentrations were also detected in NND-Dem, AD, DLB/PDD, FTD, and VaD compared to HC. Both biomarkers discriminated CJD from non-CJD dementias with an AUC of 0.93. In CJD, plasma t-tau, but not Nfl, was associated with *PRNP* codon 129 genotype and CJD subtype. Positive correlations were observed between plasma Nfl and t-tau concentrations, as well as between plasma and CSF concentrations of both biomarkers (*p* < 0.001). Nfl was increased in rapidly progressive AD (rpAD) compared to slow progressive AD (spAD) and associated to Mini-Mental State Examination results. However, Nfl displayed higher accuracy than t-tau discriminating CJD from rpAD and spAD. Finally, plasma t-tau, but not plasma Nfl, was significantly associated with disease duration, offering a moderate survival prediction capacity.

**Conclusions:**

Plasma Nfl and t-tau are useful complementary biomarkers for the differential diagnosis of CJD. Additionally, plasma t-tau emerges as a potential prognostic marker of disease duration.

**Supplementary Information:**

The online version contains supplementary material available at 10.1186/s13195-021-00815-6.

## Background

Neurodegenerative dementias are a group of clinically heterogeneous diseases characterized by gradual progression of cognitive dysfunction, psychiatric and behavioral symptoms, and movement deficits. They can be associated either with the aggregation and accumulation of misfolded proteins (i.e.*,* Alzheimer’s disease (AD), fronto-temporal dementia (FTD), dementia with Lewy bodies (DLB), Parkinson’s disease dementia (PDD), and Creutzfeldt-Jakob disease (CJD)) or with brain damage due to impaired blood flow, leading to vascular dementia (VaD).

The presence of overlapping symptomatology in neurodegenerative dementias is frequent, thus differential diagnosis, currently based on clinical evaluation and biological and topological markers, may be challenging [1–3]. Cerebrospinal fluid (CSF)-based tests for total-tau (t-tau), phospho-tau (p-tau) and amyloid β (Aβ42) are included in the diagnostic criteria of AD [4], while 14-3-3 protein and PrP^Sc^ detection by the real-time quacking induced conversion (RT-QuIC) are included in the diagnostic criteria of CJD [5, 6]. Other CSF biomarkers such as neurofilament light (Nfl) are increased in the CSF of all neurodegenerative dementias studied, as well as in motor neuron diseases, being considered as a general marker of neurodegeneration. Its low specificity undermines its potential use in the differential diagnostic context, but favors its use to identify and grade, or exclude, neurodegeneration. In addition, the observation that Nfl is associated to survival and disease severity in many neurodegenerative conditions suggests a potential role as dynamic and prognostic marker [7–9].

Blood-based biomarkers offer important advantages over CSF-based biomarkers. In contrast to lumbar puncture, blood collection is time- and cost-effective and can be easily obtained in primary care. Thus, development of blood-based assays may lead to the implementation of non-invasive front-line tests for early diagnosis, screening of populations, and follow-up analysis of patients (disease monitoring). Despite the potential advantages of blood biomarkers, the low concentrations of brain-derived biomarkers in blood impeded their study and validation as robust biomarkers until the recent development of antibody-based ultrasensitive technologies [10]. In this regard, alterations of brain biomarkers in blood have been recently reported in several neurodegenerative dementias. Nfl and t-tau are two of the most promising ones to be translated into clinical grounds regarding their diagnostic and prognostic value, especially in CJD, where both proteins are highly increased compared with controls and other neurodegenerative diseases [11–16]. Since Nfl and t-tau are associated with neuronal damage, a common hallmark in neurodegenerative dementias, it is crucial to determine their specificity and accuracy in the differential diagnostic context.

The main objective of this study was to validate previous observations on the diagnostic accuracy of plasma Nfl and t-tau in CJD with additional consideration of relevant non-neurodegenerative and neurodegenerative differential diagnoses such as rapidly progressive AD (rpAD). Further, we investigated plasma Nfl and t-Tau in CJD-subtypes, associations with other known CJD-biomarkers, influence of disease stage, and prognostic values. Finally, we compared the diagnostic and prognostic accuracy of both biomarkers between plasma and CSF.

## Methods

### Study design, population, and data acquisition

Data and samples were collected in the framework of a prospective study on CJD surveillance and diagnostics. For this retrospective analysis, we utilized the clinical database and the biobank of the German National Reference Center for Transmissible Spongiform Encephalopathies. CJD cases were selected on the base of availability of plasma samples, clinical information, and sufficient diagnostic characterization. The clinical and demographic information had been recorded during the diagnostic process through a standardized questionnaire including a third-party anamnesis. Samples from neurological disease control groups and neurodegenerative dementias were obtained at the Department of Neurology of the University Medical Center and the National Reference Center for Creutzfeldt-Jakob disease and healthy controls (HC) at the Department of Transfusion Medicine, University Medical Center Göttingen (Germany). A total of 309 plasma samples were used in this study. Blood was collected in EDTA tubes and centrifuged at 1500×*g* and 4 °C for 10 min under same pre-analytical conditions. CSF sampling in CJD cases was performed at the same day as blood uptake or up to a maximum of 15 days earlier.

### Case and sample characterization

The healthy control (HC) group was composed of healthy blood donors with absence of any relevant clinical findings. The neurological disease control group was composed of cases diagnosed with neurological conditions either without (NND) or with cognitive impairment or dementia at the time of sampling (NND-Dem). NDD-Dem cases were initially suspected of CJD (“CJD-mimics”) but prion diseases were subsequently excluded. NND and NND-Dem cases were diagnosed according to acknowledged standard neurologic clinical and para-clinical findings based on the ICD 10 definitions. The NND group included the following diagnostic groups: epilepsy, psychiatric disorders, headache, hypoxia, cerebral lymphoma, paraneoplasia, vertigo, vascular encephalopathy, and pain syndromes, while the NND-Dem group included cerebral vasculitis, normal-pressure hydrocephalus, Wilson’s disease, CNS neoplasia, encephalitis, ischemic stroke, and dementia due to alcohol abuse. Alzheimer’s disease (AD) was diagnosed according to the National Institute on Aging - Alzheimer’s Association workgroups (NIA-AA) criteria [4]. Stratification of AD cases in slow progressive AD (spAD), and rapid progressive AD (rpAD) was based on rate of cognitive decline. Rapid progression was defined by a cognitive decline of more than 6 points per year on the Mini Mental Status Examination (MMSE) scale. Cases with no rapid progression 1 year before or after blood collection were classified as rpAD. Velocity of decline was calculated using linear regression (least square method) as described before [17]. Sporadic Creutzfeldt-Jakob disease (CJD) cases were diagnosed according to consensus criteria in either probable (clinical diagnosis, *n* = 15) or definite (neuropathological confirmation, *n* = 68) [6]. Diagnosis of dementia with Lewy bodies (DLB) was based on the criteria of McKeith [18]; Parkinson’s disease dementia (PDD) diagnosis was based on the task force of the Movement Disorder Society criteria [19] and differentiated from other Parkinson-plus syndromes using established diagnostic criteria for corticobasal degeneration [20], progressive supranuclear palsy [21], and multiple system atrophy [22]. Fronto-temporal dementia (FTD) was diagnosed according to the International Behavioural Variant FTD Criteria Consortium for bvFTD [23]. Vascular dementia (VaD) diagnosis was based on clinical and radiological criteria as described by the (National Institute of Neurological and Communicative Disorders and Stroke and the Alzheimer’s Disease and Related Disorders Association) [24]. Relevant co-pathologies were excluded in all diagnostic groups by clinical criteria and review of the records from the diagnostic work-up, including MRI scans. In the VaD group, CSF p-Tau and beta-amyloid 1-42 were considered to exclude concomitant AD pathology as far as possible.

To calculate the influence of sampling and disease severity in biomarkers concentrations, CJD cases were stratified in three categories according to whether blood was collected in the first, second, or third tertial of the total disease duration. Additionally, CJD cases were classified as early stage, in which at least one clinical hallmark for CJD [6] but no complete loss of communication ability and voluntary movement was present, and late stage, akinetic mutism. To evaluate the prognostic value of plasma Nfl and t-tau as prognostic markers, disease duration was recorded as the time (in days) from symptom onset or from blood uptake to the death of the patient. Symptom onset was evaluated through a third-party questionnaire (wife, spouse, or 1st grade relative) and defined as the date either cognitive, visual, balance, or movement disturbances had become apparent.

### Plasma and CSF tests

Plasma Nfl and total tau levels were measured using commercially available kits on the Single molecule array (Simoa) HD-1 Analyzer (Quanterix). YKL-40 was measured using the MicroVueYKL-40 EIA ELISA kit from Quidel as previously described [25]. Total-Prion protein (t-PrP) was quantified using a fluorometric custom-made ELISA as described before [26]. CSF Nfl and total-tau (t-tau) were quantified using the enzyme-linked immunosorbent assay kits NF-light (UmanDiagnostics) and INNOTEST hTAU-Ag (Fujirebio), respectively. CSF was analyzed for the presence of 14-3-3 protein using western blot [27]. Amyloid β (Aβ42) was measured using the INNOTEST® β-AMYLOID ELISA kit from Fujirebio.

### Statistical analysis

Comparison of mean age between diagnostic groups was performed with ANOVA-test and Tukey correction. Differences in the sex ratio were compared with chi-squared test, and *p* values were adjusted with the Holm method. Comparison of biomarker levels among diagnostic groups was performed with linear regression models. Biomarker data were log-transformed, and age and sex were included as covariates. Multiple comparisons of means were performed with Tukey contrasts, available in the multcomp R package [28]. The same analysis was applied in the investigation of the influence of the *PRNP* codon 129 genotype and the time of sampling on biomarkers levels. Comparison of biomarker levels in 14-3-3 positive and negative groups was performed using the Mann-Whitney test. Spearman rank coefficients were used to quantify associations between continuous biomarkers levels. To assess the diagnostic accuracy of plasma Nfl and t-tau, receiver operating characteristic (ROC) curve analyses were carried out and areas under the curve (AUC) with 95% confidence intervals (95% CI) were calculated. AUC values were compared with pROC R package, using the Bootstrap method [29]. The best cutoff values were estimated based on the Youden index. The CJD cases were stratified in two groups based on the duration of the disease: short course (when disease duration from onset was below mean disease duratio*n* = 165 days) or long course (otherwise). Differences on biomarker levels between these two groups were assessed with linear regression models controlling for demographic covariates where the biomarker data were log transformed. Relationship between disease duration and each biomarker was explored with the non-parametric Spearman correlation coefficient and with Cox proportional hazards (PH) models controlling for significant covariates (age, sex, and *PRNP* codon 129 genotype), using the survival R package [30]. PH assumption was tested with the Schoenfeld residuals against the transformed time. To allow for non-linear associations between biomarker data and disease duration, we employed the multivariable fractional polynomial method, using the mfp package in R [31]. Graphical representation of regression models was performed with visreg R package [32]. Statistical significance was considered at *p* < 0.05.

## Results

### Plasma Nfl and t-tau in controls, CJD, and differential diagnoses

Data on age, sex, and plasma biomarkers for the study cohort are presented in Table 1. No significant differences on age were detected between diagnostic groups. Statistically significant differences in the sex ratio were detected in CJD compared to DLB/PDD (*p* = 0.04) and to HC (*p* = 0.0016). In HC, mean values of Nfl differed between sexes (female = 8.03 pg/mL, male = 9.75 pg/mL) with borderline significance (*p* = 0.044) whereas no significant differences were detected for Nfl in CJD and for t-tau in both groups.

**Table 1:** Demographic and biomarkers data from the study population

	n	Sex (f/m)	Age (years)	Duration (days)*	Plasma Nfl (pg/mL)		Plasma t-Tau (pg/mL)	
					Mean + SD	95% CI	Mean + SD	95% CI
**Controls**								
HC	70	22/48	64.7 ± 5.1	–	9.2 ± 3.4	8.4–10.0	2.5 ± 1.2	2.3–2.8
NND	26	13/13	64.1 ± 6.9	–	23.4 ± 39.6	7.4–39.5	3.7 ± 2.4	2.7–4.7
NND-Dem	17	9/8	63.2 ± 16.9	–	182.2 ± 151.5	104.3–260.1	3.1 ± 2.4	1.8–4.3
**Neurodegenerative and vascular dementia**								
AD	44	26/18	68.5 ± 10.9	–	34.9 ± 33.4	24.7–45.8	3.6 ± 2.4	2.8–4.3
CJD	83	53/30	66.6 ± 8.8	273.4 ± 276.9	349.7 ± 505.4	239.4–460.1	45.1 ± 48.7	34.5–55.7
MM	51	36/16	66.8 ± 9.6	236.9 ± 213.7	384.5 ± 492.7	246.0–523.1	59.8 ± 54.4	44.6–75.1
MV	11	4/7	65.6 ± 7.1	316.3 ± 20.1	174.9 ± 118.4	95.3–254.4	23.7 ± 24.9	6.9–40.4
VV	18	11/7	68.9 ± 6.7	369.5 ± 417.3	395.1 ± 689.4	52.3–737.9	16.5 ± 19.3	6.9–26.2
DLB/PDD	35	11/24	69.8 ± 8.3	–	46.8 ± 37.1	33.8–59.7	2.3 ± 1.4	1.8–2.7
FTD	12	8/4	69.5 ± 7.2	–	27.8 ± 29.6	7.9–47.8	2.3 ± 1.3	1.4–3.1
VAD	22	15/5	70.0 ± 9.9	–	56.35 ± 51.7	33.4–79.3	3.2 ± 2.8	2.0–4.8

Number of cases studied, sex (number of female and male cases), age at onset (mean age with SD), and plasma Nfl and t-tau (mean concentrations with SD and 95% CI) are indicated. Information on disease duration (onset to death, mean days, SD) is only available for the CJD group. HC, healthy controls; NND, neurological diseases without dementia; NND-Dem, neurological diseases with dementia; AD, Alzheimer’s disease; CJD, Creutzfeldt-Jakob disease; MM/MV/VV, PRNP codon 129 methionine/valine polymorphism; DLB/PDD, dementia with Lewy bodies/Parkinson’s disease dementia; FTD, fronto-temporal dementia; VaD, vascular dementia; Nfl, neurofilament light; t-tau, total-tau. *Information on disease duration was available from 82 of 83 CJD cases

**Fig. 1:**
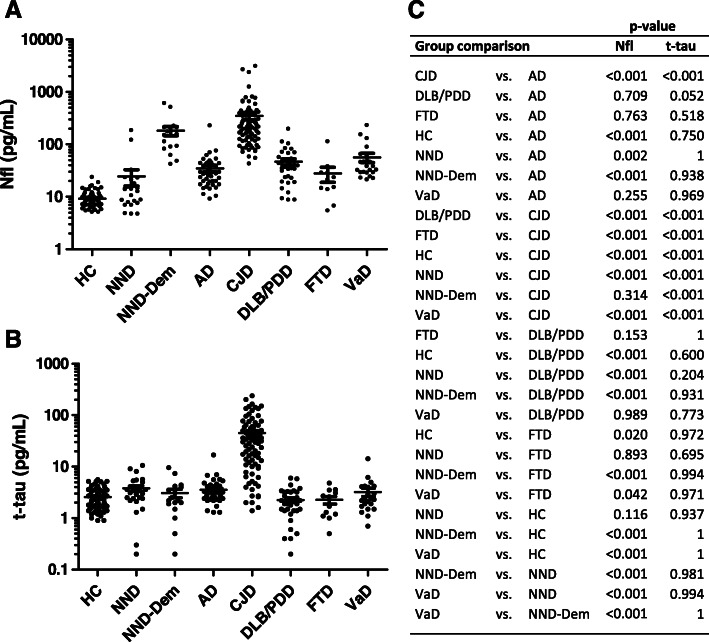
Plasma Nfl and t-tau in the differential diagnosis of neurodegenerative dementia. Dot plot displaying **a** Nfl and **b** t-tau concentrations in the differential diagnosis of neurodegenerative dementia. **c** Statistical significance derived from a multi-comparative analysis corrected for covariates for Nfl and t-tau among the diagnostic groups (linear regression models were used, as explained in the “Statistical analysis” section). The Tukey-corrected *p* values for each pair of diagnostic group comparison are indicated. HC, healthy controls; NND, neurological diseases without dementia; NND-Dem, neurological diseases with dementia; AD, Alzheimer’s disease; CJD, Creutzfeldt-Jakob disease; DLB/PDD, dementia with Lewy bodies/Parkinson’s disease dementia; FTD, fronto-temporal dementia; VaD, vascular dementia; Nfl, neurofilament light; t-tau, total-tau; f, female; m, male; SD, standard deviation

The highest Nfl concentrations were detected in CJD followed by NND-Dem, VaD, DLB/PDD, AD, FTD, NND, and HC (Table 1 and Fig. 1a). In a multi-comparative analysis corrected for covariates, Nfl concentrations were increased in CJD compared to HC, NND, AD, DLB/PDD, FTD, and VaD (*p* < 0.001), in AD compared to HC (*p* < 0.001) and NND (*p* = 0.002), in DLB/PDD compared to HC and NND (*p* < 0.001), in FTD compared to HC (*p* = 0.020), and in VaD compared to HC and NND (*p* < 0.001). Additionally, significantly higher Nfl concentrations were observed in NND-Dem compared to HC, NND, AD, DLB/PDD, FTD, and VaD (*p* < 0.001) (Fig. 1a, c). In contrast, t-tau was exclusively increased in CJD, with significantly different concentrations when compared to HC, NND, NND-Dem, AD, DLB/PDD, FTD, and VaD (*p* < 0.001) (Fig. 1b, c).

The diagnostic accuracy of Nfl and t-tau discriminating the different diagnostic groups was calculated using the areas under the curve (AUC) and 95% confidence intervals (95% CI) (Table 2). Nfl was able to differentiate HC and NND from neurodegenerative dementias with good accuracy (AUC > 0.81 in all cases, with the exception of the NND vs. FTD comparison, AUC = 0.66). Nfl displayed a remarkable discriminatory value for CJD compared with non-demented controls (AUC = 1, vs. HC and AUC = 0.97 vs. NND). High Nfl levels in NND-Dem were translated in AUC values ranging from 0.88 to 0.97 discriminating AD, DLB/PDD, FTD, and VaD cases and lower accuracy discriminating from CJD cases (AUC = 0.67). In contrast, t-tau exclusively displayed a good discriminatory value in the differentiation of CJD from HC, NND, and NND-Dem (AUCs values ranging from 0.91 to 0.95). Significant differences in AUC values between Nfl and t-tau for each comparison indicates high specificity for t-tau in the discrimination of CJD cases from the other diagnostic groups, while Nfl resulted in a less specific test with a good diagnostic value in the discrimination of all conditions associated to dementia from HC and NND (Fig. 1c, Table 2). Overall, Nfl presented a slightly superior, but not significant, accuracy compared to t-tau in discriminating CJD from neurodegenerative dementias, with the exception of the AD vs. CJD comparison where Nfl significantly over performs t-tau (Table 2).

**Table 2:** Diagnostic value of plasma Nfl and t-tau in the differential diagnosis of neurodegenerative dementias

	Nfl		t-tau		
	AUC	95% CI	AUC	95% CI	***p*** value
HC vs. AD	0.97	0.94–0.99	0.67	0.57–0.77	**< 0.001**
HC vs. CJD	1	1	0.95	0.91–0.98	**0.003**
HC vs. DLB/PDD	0.95	0.91–0.99	0.57	0.46–0.70	**< 0.001**
HC vs. FTD	0.81	0.61–1	0.60	0.42–0.80	0.1
HC vs. VaD	0.99	0.99–1	0.55	0.39–0.67	**< 0.001**
NND vs. AD	0.82	0.70–0.93	0.52	0.37–0.67	**< 0.001**
NND vs. CJD	0.97	0.94–1	0.91	0.86–0.96	**0.05**
NND vs. DLB/PDD	0.84	0.72–0.95	0.71	0.57–0.85	0.1
NND vs. FTD	0.66	0.46–0.87	0.69	0.52–0.87	0.7
NND vs. VaD	0.90	0.80–1	0.62	0.45–0.78	**0.002**
NND-Dem vs. AD	0.95	0.90–1	0.62	0.44–0.80	**< 0.001**
NND-Dem vs. CJD	0.67	0.53–0.80	0.93	0.89–0.98	**< 0.001**
NND-Dem vs. DLB/PDD	0.91	0.83–0.99	0.56	0.42–0.77	**0.001**
NND-Dem vs. FTD	0.97	0.90–1	0.58	0.36–0.79	**< 0.001**
NND-Dem vs. VaD	0.88	0.78 0.99	0.51	0.32–0.69	**0.002**
AD vs. CJD	0.98	0.96–1	0.91	0.87–0.96	**0.01**
AD vs. DLB/PDD	0.62	0.49–0.75	0.72	0.61–0.84	0.3
AD vs. FTD	0.66	0.46–0.85	0.70	0.51–0.88	0.7
AD vs. VaD	0.69	0.56–0.82	0.63	0.48–0.79	0.6
CJD vs. DLB/PDD	0.96	0.93–0.99	0.96	0.92–0.99	0.7
CJD vs. FTD	0.98	0.95–1	0.96	0.92–0.99	0.2
CJD vs. VaD	0.94	0.88–0.99	0.93	0.88–0.98	0.8
DLB/PDD vs. FTD	0.72	0.54–0.90	0.51	0.30–0.70	0.08
DLB/PDD vs. VaD	0.47	0.37–0.68	0.60	0.45–0.75	0.3
FTD vs. VaD	0.82	0.65–0.99	0.63	0.40–0.82	0.1

AUC derived from ROC curves, with 95% CI in the comparative analysis of HC, NND, and NND-Dem vs. neurodegenerative dementia and between the different neurodegenerative dementia groups are indicated. Statistical differences (*p* values) between AUC values for Nfl and t-tau in the comparisons between pairs of diagnostic groups calculated as explained in the “Statistical analysis” section. AUC, area under the curve; ROC, receiver operating characteristic; 95% CI, 95% confidence interval; HC, healthy controls; NND, neurological diseases without dementia; NND-Dem, neurological diseases with dementia; AD, Alzheimer’s disease; CJD, Creutzfeldt-Jakob disease; DLB/PDD, dementia with Lewy bodies/Parkinson’s disease dementia; FTD, fronto-temporal dementia; VaD, vascular dementia; Nfl, neurofilament light; t-tau, total-tau

Plasma Nfl and t-tau displayed a positive and significant association in the NND-Dem (cc: 0.6957, *p* = 0.0019) and CJD (cc: 0.3676, *p* < 0.001) groups (Additional file 1A and 1B), while no other significant correlations were detected in the rest of the diagnostic groups (*p* > 0.05). In CJD, a significant positive correlation was observed between plasma and CSF Nfl concentrations (cc: 0.5125, *p* < 0.001) (Additional file 1C) and between plasma and CSF t-tau concentrations (cc: 0.5425, *p* < 0.001) (Additional file 1C). Plasma Nfl was associated with CSF t-tau (cc: 0.3266, *p* = 0.0109), but plasma t-tau was not associated with CSF Nfl. Plasma t-tau was associated with CSF 14-3-3 positivity (*p* = 0.0211). Plasma Nfl and t-tau were associated neither with CSF Aβ42 nor with CSF t-PrP. Plasma Nfl, but not t-tau, significantly correlated with plasma YKL-40 (cc: 0.4030 *p* = 0.0081) (Additional file 1C).

### Plasma Nfl and t-tau cutoff points for the differential diagnosis of CJD

Cutoff points and associated sensitivity and specificity values for the discrimination of CJD from HC and non-CJD-Dem (NND-Dem, AD, FTD, DLB/PDD and VaD) were determined. Due to the significant differences on Nfl concentrations between AD, FTD, DLB/PDD, VaD and NND-Dem, an additional group including only neurodegenerative dementias (AD, FTD, DLB/PDD and VaD) named non-CJD neurodeg-Dem was defined and included in the analysis. At 33 pg/mL Nfl cutoff, CJD was discriminated from HC with 100% sensitivity and specificity. At 70 pg/mL Nfl cutoff, CJD was discriminated from non-CJD-Dem with 79% sensitivity and 96% specificity and from non-CJD neurodeg-Dem (excluding NND-Dem) with 88% sensitivity and 96% specificity (Table 3). At 6.0 pg/mL t-tau cutoff, CJD was discriminated from HC with 84% sensitivity and 100% specificity. At 6.1 pg/mL t-tau cutoff, CJD discriminated from non-CJD-Dem and non-CJD neurodeg-Dem with 95% sensitivity and 84% (Table 3).

**Table 3:** Diagnostic accuracy of plasma Nfl and t-tau in the discrimination of CJD

	Plasma Nfl			Plasma t-tau		
CJD vs.	Cutoff	Sensitivity (%)	Specificity (%)	Cutoff	Sensitivity (%)	Specificity (%)
HC	> 33 pg/mL	100	100	> 6.0 pg/mL	84	100
Non-CJD-Dem	> 70 pg/mL	79	96	> 6.1 pg/mL	95	84
Non-CJD-neurodeg-Dem	> 70 pg/mL	88	96	> 6.1 pg/mL	95	84

Sensitivity (in %), specificity (in %), and associated cutoff points (in pg/mL) for plasma Nfl and t-tau in the discrimination of CJD from HC, non-CJD-Dem, and non-CJD neurodeg-Dem

### Plasma Nfl and t-tau in the differential diagnosis of CJD from AD subtypes

**Fig. 2:**
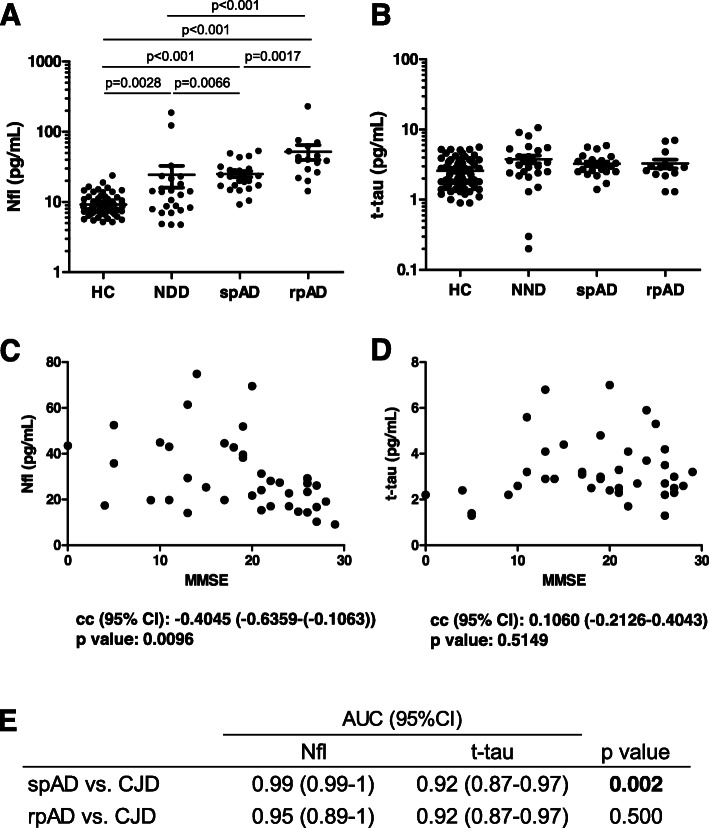
Plasma Nfl and t-tau in different AD subtypes and discrimination from CJD. Dot plot displaying **a** Nfl and **b** t-tau concentrations in HC (*n* = 70), NND (*n* = 26), spAD (*n* = 24), and rpAD (*n* = 16). Statistical significance derived from a comparative analysis corrected for covariates is indicated. Correlation analysis between plasma Nfl (**c**) and t-tau (**d**) concentrations with MMSE in AD cases. Correlation coefficients with 95% CI and associated *p* values derived from Spearmen test analysis are indicated. **e** AUC derived from ROC curves, with 95% CI in the comparative analysis of spAD and rpAD from CJD cases. *p* values derived from the comparative analysis of AUCs are indicated (corresponding statistical test is explained in Statistical analysis). AD, Alzheimer’s disease; spAD, slow progressive AD; rpAD, rapid progressive AD; Nfl, neurofilament light; t-tau, total-tau; AUC, area under the curve; ROC, receiver operating characteristic; 95% CI, 95% confidence interval

Rapid progressive forms of AD have been widely described, being one of the main differential diagnosis of CJD due to their partial overlap on clinical presentation and biomarker profile [33]. Thus, we examined whether different Nfl and t-tau profiles were observed between slow progressive (spAD) and rapid progressive (rpAD) AD cases. Plasma Nfl displayed significantly higher concentrations in rpAD (52.1 ± 49.6 pg/mL) than in spAD (25.5 ± 11.7 pg/mL) (Fig. 2a), with an associated AUC of 0.79 (95%CI = 0.64–0.93). In contrast, no differences in t-tau concentrations were observed between rpAD (3.3 ± 1.7 pg/mL) and spAD (3.3 ± 1.1 pg/mL) (Fig. 2b), with an associated AUC of 0.57 (95%CI = 0.37–0.77). The higher Nfl concentrations in rapid progressive forms of AD were in agreement with a significant negative correlation between Nfl and MMSE score (Fig. 2c), an association that was not observed for t-tau (Fig. 2d). To investigate this further and to exclude that the plasma Nfl differences between spAD and rpAD are solely associated with lower MMSE scores in the rpAD group, we performed an additional linear regression model. When age, sex, and MMSE scores were included, higher Nfl concentrations still showed a significant association with rpAD (*p* = 0.028). Nfl had a higher accuracy than t-tau discriminating CJD from spAD (Nfl AUC = 0.99 vs. t-tau AUC = 0.95) and CJD from rpAD (Nfl AUC = 0.95 vs. t-tau AUC = 0.92). However, differences between AUCs were only significant for the spAD vs. CJD comparison (Fig. 2e).

### Influence of codon 129 *PRNP* genotype and CJD subtype

**Fig. 3:**
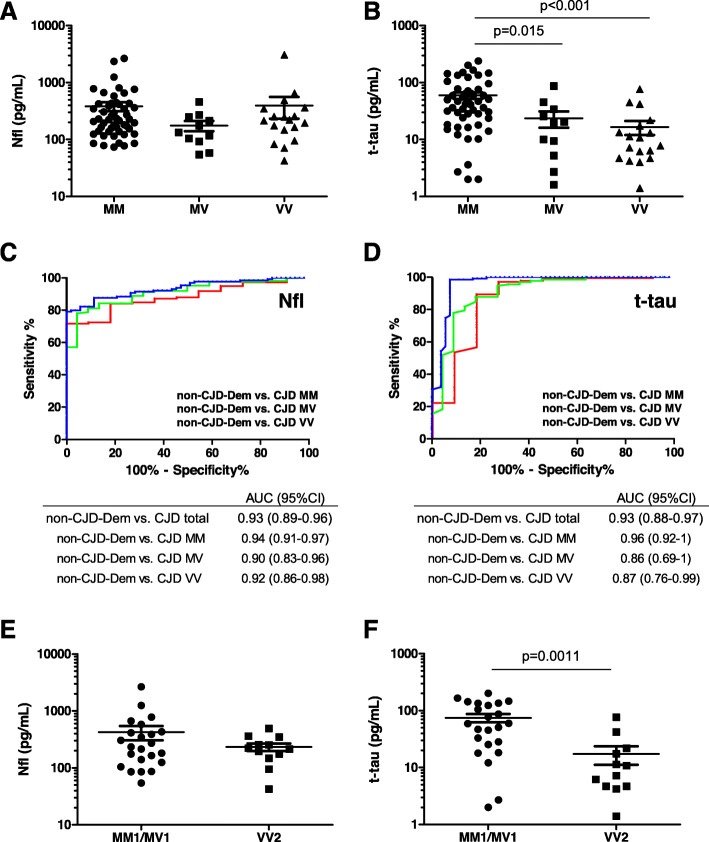
Influence of codon 129 *PRNP* genotype and subtype in plasma Nfl and t-tau in CJD. **a** Nfl and **b** t-tau concentrations in CJD stratified by prion protein gene (*PRNP*) codon 129 genotype (MM, *n* = 51; MV, *n* = 11; VV, *n* = 18). Statistical significance derived from a multi-comparative analysis corrected for covariates for Nfl and t-tau is indicated (linear regression models were used, as explained in the “Statistical analysis” section). Dot plots with mean (line) values are shown. ROC curves for Nfl (**c**) and t-tau (**d**) in the comparative analysis of non-CJD-Dem vs. CJD cases stratified by codon 129 *PRNP* genotype. AUC derived from ROC curves, with 95% CI in the comparative analysis of non-CJD-Dem vs. total CJD cases and CJD cases stratified by codon 129 *PRNP* genotype are shown. **e** Nfl and **f** t-tau concentrations in CJD MM1/MV1 (*n* = 23) and VV2 (*n* = 12) subtypes. Statistical significance derived from a comparative analysis corrected for covariates is indicated. Dot plots with mean (line) values are shown. Non-CJD-Dem, non-neurodegenerative dementia; Nfl, neurofilament light; t-tau, total-tau

To further investigate the role of different CJD subtypes in plasma Nfl and t-tau concentrations, CJD cases were stratified according to codon usage (Methionine (M) or Valine (V)) at position 129 of the *PRNP* gene. It determines the clinico-pathological features of the disease [34] and is a well-known modifier of Nfl and t-tau accuracy in biological fluids in prion diseases [7, 14, 15, 35]. In a multi-comparative analysis corrected for covariates, no significantly different Nfl concentrations were detected between MM (384.5 ± 492.7 pg/mL), MV (174.9 ± 118.4 pg/mL), and VV (395.1 ± 689.4 pg/mL) cases (Fig. 3a). In contrast, t-tau concentrations were significantly higher in MM (59.4 ± 54.4 pg/mL) compared to MV (23.6 ± 24.9 pg/mL) and to VV (16.5 ± 19.3 pg/mL) cases (Fig. 3b). Given the influence of codon 129 *PRNP* genotype on Nfl and t-tau profiles in CJD cases, we sought to determine the diagnostic performance of both biomarkers in the discrimination of CJD from neurodegenerative dementias from a non-prion etiology. To this purpose, different types of dementias (NND-Dem, AD, DLB/PDD, FTD, and VaD) were grouped under the non-CJD dementia label (non-CJD-Dem), and AUC values calculated for CJD cases stratified by codon 129 genotype. In total CJD cases, equal AUCs were obtained for Nfl and t-tau in the discrimination of non-CJD-Dem (AUC = 0.93) (Fig. 3c, d). Stratification by *PRNP* codon 129 genotype had a stronger influence in t-tau (AUCs ranging from 0.86 to 0.96), than in Nfl (AUCs ranging from 0.90 to 0.94).

Regarding CJD subtype, data were available for the two most prevalent subtypes. While no differences were detected in Nfl concentrations between MM1/MV1 (423.1 ± 561.9 pg/mL) and VV2 (233.6 ± 122.9 pg/mL) cases (Fig. 3e), t-tau was significantly increased in MM1/MV1 (75.0 ± 58.2 pg/mL) compared to VV2 (17.4 ± 21.7 pg/mL) cases (Fig. 3f).

### Relation with disease stage and prognostic value of plasma Nfl and t-tau in CJD

**Fig. 4:**
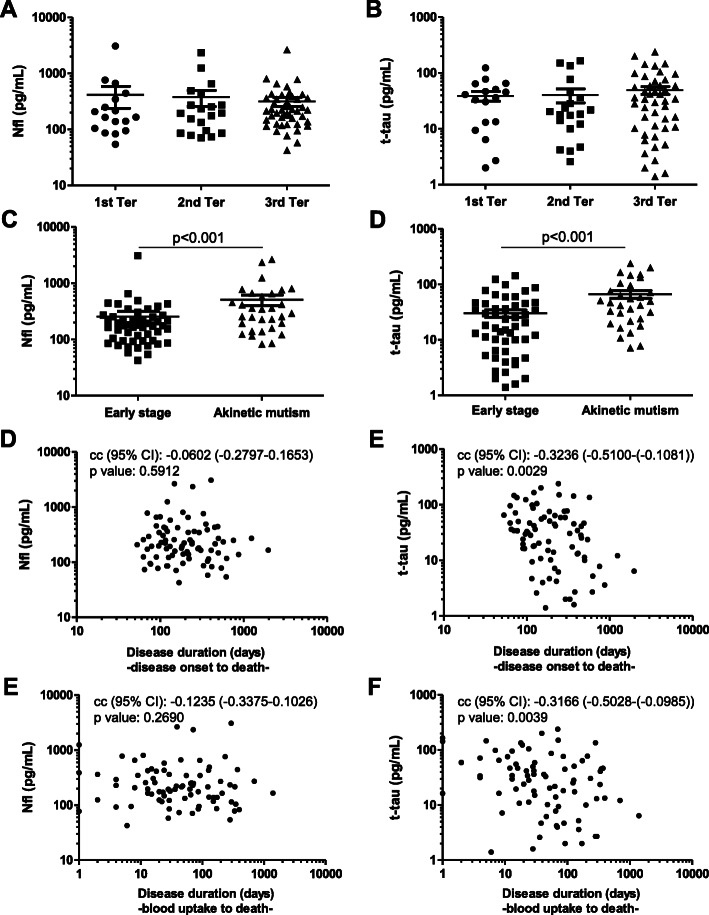
Relation with disease stage and prognostic value of plasma Nfl and t-tau in CJD. Plasma Nfl (**a**) and t-tau (**b**) concentrations in CJD cases stratified in three categories according to whether blood was collected in the first stage/tertial (1st Ter, *n* = 17) (time of blood uptake to disease onset/total duration of the disease < 0.33), second stage/tertial (2nd Ter, *n* = 20) (0.33–0.66), or third stage/tertial (3rd Ter, *n* = 46) (> 0.66) of the disease. Plasma Nfl (**c**) and t-tau (**d**) in CJD cases classified as early stage (*n* = 51) and akinetic mutism (*n* = 31). Resultant significant corrected *p* values are displayed. Nfl, neurofilament light; t-tau, total-tau. **d**–**f** Association between disease duration (considered from disease onset and from blood uptake) and plasma Nfl and t-tau, measured with Spearman correlation coefficients (cc) and corresponding *p* values

To evaluate a potential association between Nfl and t-tau levels at the time of blood collection and the timeliness of the disease in CJD patients, samples were stratified in early (1st Ter), middle (2nd Ter) and late stages (3rd Ter). However, neither Nfl nor t-tau concentrations were significantly different between disease stages (Fig. 4a, b). Next we stratified cases according whether patients presented early symptoms or akinetic mutism at the time of sampling (as explained in Study Population). Cases in akinetic mutism showed significantly higher Nfl and t-tau levels than those at early stage of the disease (Fig. 4c, d).

To ascertain whether plasma Nfl and t-tau were associated to disease duration, CJD cases were stratified in those displaying short and long course (lower and higher than mean disease duration, respectively). Nfl concentrations were not significantly different between CJD cases displaying short and long survival times. In contrast, higher t-tau concentrations were detected in CJD cases with short survival time (*p* = 0.0088). Association between disease duration and biomarker data was explored with Spearman correlation coefficients. Due to the difficulty to precisely define disease duration in prion diseases, in our study, we considered two starting points: from disease onset to death and from blood uptake to death. Significant associations were observed in the case of t-tau (cc = − 0.3236 and cc = − 0.3166, when disease duration was considered from disease onset and blood uptake respectively), but not in the case of Nfl (Fig. 4d–f). Given these results, we built Cox PH models with t-tau and Nfl as predictors to observe the effect over disease duration. As expected, only t-tau behaved as a significant predictor, although with very modest hazard ratios (HR), which is not surprising considering the unit used to measure the biomarker (pg/mL) (Additional file 2). Non-linear relationships between disease duration and plasma t-tau were also explored (Additional file 2), but the best models displayed only moderate fitting, with concordance indices of 0.6 (i.e., 60% of concordant prediction-value pairs) [36].

### Comparative analysis of plasma and CSF accuracy in CJD diagnosis and prognosis

**Fig. 5:**
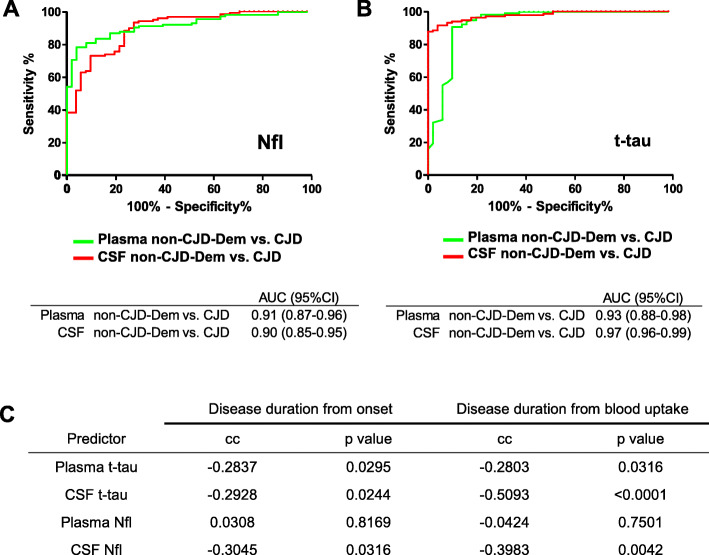
Comparative analysis of plasma and CSF biomarkers accuracy in CJD diagnosis and disease duration. **a**, **b** ROC curves with associated AUC values in the comparative analysis of CJD (*n* = 51) vs. non-CJD-Dem (*n* = 118) groups obtained with t-tau and Nfl in CSF and plasma. **c** Association of plasma and CSF t-tau and Nfl with disease duration measured with Spearman correlation coefficients (cc) and corresponding *p* values

In order to comparatively evaluate the accuracy of plasma and CSF Nfl and t-tau, both biomarkers were measured in available paired CSF cases in CJD and neurodegenerative dementia (non-CJD-Dem) cases. Plasma and CSF Nfl showed a similar accuracy in discriminating non-CJD-Dem from CJD (plasma AUC = 0.91 and CSF AUC = 0.90) (Fig. 5a). In contrast, CSF t-tau (AUC = 0.97) displayed superior accuracy than plasma t-tau (AUC = 0.93) in the same comparative analysis (Fig. 5b). Association between biomarkers and disease duration was explored with Spearman correlation coefficient to compare the potential prognostic value of CSF vs. plasma markers in the group of available paired cases. While both CSF t-tau and Nfl levels were strongly inversely associated with disease duration, particularly when this was measured from blood uptake (cc = − 0.5093 and cc = − 0.3983 for CSF t-tau and CSF Nfl, respectively), only plasma t-tau showed association with disease duration (cc = − 0.2803). As also observed in the previous section, plasma Nfl totally lacked of prognostic value (Fig. 5c).

## Discussion

### Plasma Nfl and t-tau in the differential diagnosis of CJD

The differential diagnosis of neurodegenerative dementia may be challenging due to overlapping clinical features among different conditions. Here, we explored the diagnostic accuracy of plasma Nfl and t-tau in the differential diagnosis of CJD, identified the influence of demographic and genetic factors on biomarker levels and determined the diagnostic value of both biomarkers in the differential diagnostic context. Our data validate previous reports describing elevated blood Nfl and t-tau in CJD compared to HC [14, 15] as well as to non-demented controls [11].

Additionally, we observed that, despite the overall accuracy of both biomarkers discriminating CJD from non-CJD dementias is similar (AUC = 0.93), t-tau concentrations are dependent on the genetic characteristics of the CJD population (codon 129 genotype). Importantly, the accuracy of both biomarkers is highly dependent to the diagnostic group to which CJD is compared. In this regard, while Nfl displays a high sensitivity discriminating CJD from non-demented controls, test specificity is hampered by the presence of high concentrations in non-CJD dementias. In contrast, t-tau was somewhat less sensitive than Nfl discriminating CJD form non-demented controls, but offers higher test specificity for CJD. This, together with the observation that Nfl concentrations are significantly higher in NND-Dem compared with non-CJD neurodegenerative dementias, discloses the complementarity of both biomarkers in the differential diagnosis of CJD. Thus, t-tau performs better than Nfl in discriminating CJD from NND-Dem, whereas Nfl becomes more useful in discriminating early CJD with non-specific symptoms from NND. For the differentiation of CJD from neurodegenerative dementias, slightly superior but not significantly higher accuracies were achieved by Nfl compared to t-tau, with the exception of the CJD vs. AD comparison for which Nfl significantly outperforms t-tau. In this regard, we also explored the value of plasma Nfl and t-tau in rpAD, which represents a recurrent alternative diagnosis in cases referred to prion disease surveillance centers due to its clinical overlap with CJD [34]. The stratification of AD according to rate of cognitive decline reveals that, in spite of the higher Nfl concentrations in rpAD compared to spAD, Nfl performs better than t-tau discriminating rpAD from CJD (AUC 0.95 vs. 0.92). Interestingly, elevated Nfl has been reported in other non-CJD rapid progressive neurological syndromes including AD and those presenting vascular, neoplastic and inflammatory alterations [14].

We report distinctive optimal cutoff points considering that the diagnostic accuracy may vary with clinically suspected etiology of differential diagnoses. An optimal cutoff for t-tau (6.1 pg/mL) displays same accuracy discriminating CJD from the rest of dementia syndromes regardless their etiology whereas Nfl sensitivity resulting from the optimal cutoff of 70 pg/mL decreases when the NND-Dem group is included, as a consequence of its above mentioned high Nfl concentrations. However, these cutoffs cannot be applied in a clinical context until they have been validated in independent cohorts. Different cutoff points have been described in the bibliography, e.g., 2.2 pg/mL for t-Tau and 44.7 pg/mL for NfL [11], but these were not comparable to the present ones as they were obtained in serum and included prion diseases from different etiologies and a mixture of non-demented and demented controls.

In agreement with previous observations, we validated the role of codon 129 genotype in t-tau concentrations in CJD [14, 15], with MM cases presenting higher t-tau concentrations than those harboring MV and VV. Importantly, knowledge about *PRNP* codon 129 genotype and the type of suspected differential diagnosis (either a non-neurodegenerative neurological disease with dementia syndrome or a neurodegenerative dementia) may be useful in determining which biomarker may display higher clinical value in the differential diagnosis of CJD. Similarly, our analyses put forward the possibility to determine distinct cutoff points for each codon 129 genotype, which will probably result in improved classification performance in the case of t-tau. Regarding autopsy-confirmed confirmed CJD cases, t-tau, but not Nfl, concentrations were significantly higher in CJD MM1/MV1 compared to VV2 cases in agreement with previous data [37], suggesting that despite positive correlations in plasma, Nfl, and t-tau reflect different (or only partially overlapping) pathological features in brain tissue. In this regard, while Nfl is mainly expressed in myelinated subcortical axons [38], t-tau displays a widely neuronal expression in the brain [39], and different strain-dependent regional neuronal vulnerability has been reported in CJD [34, 40]. Additionally, differences can be attributed to differential entrance and stability of both proteins in the bloodstream.

Because blood collection is performed at different stages of the disease, we analyzed the influence of the time of sampling in the biomarker levels in CJD. Cases in akinetic mutism at the time of sampling had higher plasma Nfl and t-tau levels than those at early stages of the disease. These data are in line with previous studies demonstrating the relationship between plasma t-tau and Nfl with disease progression in CJD [41]. In contrast, no differences were found between time points during disease course (tertials), potentially due to heterogeneous application of life-extending treatment or earlier blood uptakes in patients with fast disease progression.

An important observation of this study is that while plasma and CSF Nfl display similar accuracies in the discrimination of CJD from non-CJD dementias, CSF t-tau outperforms plasma t-tau, as well as plasma and CSF Nfl. This is in agreement with previous reports showing highest accuracies for CSF t-tau comparing CJD vs. non-CJD cases among the four biomarker combinations [11, 37]. The observation that Nfl and t-tau exclusively correlate in NND-Dem and CJD groups, both displaying extremely high Nfl values, may help to shed some light on the different clearance pathway of both proteins from plasma. We hypothesize that when moderate neuronal damage occurs, different clearance or turnover mechanisms lead to an absence of correlation between both biomarkers. Instead, when massive neuronal damage occurs, this effect is masked by massive accumulation of both proteins in plasma leading to a positive correlation between both proteins. Whether blood concentrations do really mirror alterations in the brain tissue needs to be carefully addressed. Some biomarkers may be expressed in other tissues or their concentrations in blood may not reflect alterations in brain due to different interchange mechanism between fluids, different modifications and forms. In this sense, our correlation analyses in paired plasma-CSF cases indicate that the plasma levels of both proteins reflect cerebral alterations.

### Plasma Nfl and t-tau in AD and VD

We also validated previous observations in AD, reporting moderately increase or no significance differences on plasma t-tau levels between AD and cognitive normal controls [42, 43]. However, while plasma t-tau lacks accuracy as a diagnostic AD marker, it is associated with an increased risk of cognitive decline in MCI [44] and AD [45] being proposed as a potential non-specific predictive biomarker of dementia [45]. Interestingly, in our AD group, plasma Nfl, but not t-tau, showed a significant association with MMSE. This is in agreement with data reporting increasing Nfl concentrations with increasing symptoms severity [46] and its correlation with CSF AD markers, hippocampal volume loss, and FDG-PET hypometabolism [47, 48]. Our analyses in VaD cases showed high levels of plasma Nfl (only lower than those of CJD and NND-Dem), which is in line with previous studies demonstrating the association of plasma Nfl and the risk of VaD [49]. By contrast, plasma t-tau was not altered in VaD. Indeed, it seems that this biomarker in case of vascular disease is only a good marker in acute cerebrovascular events [50].

### Prognostic value of plasma Nfl and t-tau in CJD

Although disease duration in CJD is strongly determined by individual features such as age at onset, sex and *PRNP* codon 129 genotype [51, 52], the potential prognostic capacity of several fluid biomarkers is under investigation, with t-tau highlighting as one of the more promising candidates [52, 53]. In the present work, we observed a strong association between disease duration and plasma t-tau in CJD, in line with previous reports [37, 53]. It was shown that the combinatory use of information on CSF t-Tau and *PRNP* codon 129 genotype has a higher prognostic value than genotype alone [52]. Our PH model included codon 129 genotype as a controlling factor and suggests that plasma t-Tau may have potential to function in a similar way. By contrast, we did not find a significant association between survival and plasma Nfl in our study cohort. This association has been previously reported but seemed to be dependent on the CJD subtype and, upon stratification of cases, plasma Nfl was only associated with survival in a group of slowly progressive prion diseases [37]. Discrepancies between our findings and previously published studies can be related to the lack of CJD stratification, which we chose not to perform since CJD subtype information is not known at the time of diagnosis. Another reason is the different definition of survival in CJD. Whereas other authors considered relevant survival time until akinetic mutism stage when life-extending treatments are applied, we strictly considered survival time until death. This issue involves ethics considerations and needs to be clarified to reach a consensus definition of survival in these prognostic analyses.

### Study strengths and limitations

The main limitations of our study are the absence of serial samples, impeding assessment of longitudinal changes in biomarker levels, and the absence of an external validation cohort, which implies bias in the reported diagnostic accuracies. We suggest that future validation studies should aim to include longitudinal samples but this might be complicated by short survival time in CJD. To resolve this problem, the intervals between blood uptakes have to be carefully thought out. Differences might be detectable even over short time periods of 2 or 4 weeks. In our study, blood and CSF sampling were performed in close temporal association. In some cases however, blood-uptake was performed up to a maximum of 15 days later than lumbar puncture. In the context of a rapidly progressive diseases, even this rather short period of time may possibly lead to an overestimation of the diagnostic accuracy of plasma markers in comparison to CSF markers. Although samples from CJD patients were initially collected in the framework of a prospective study, case selection was done retrospectively, based on availability of sufficient information and biomaterial. Naturally, this comes along with potential biases such as the possibility that the study cohort might not exactly reflect the phenotypical spectrum of CJD at diagnosis in the population. On the other hand, the majority of CJD subtypes and *PRNP* Codon 129 Genotypes were MM/MV1 and MM, respectively, similar to what is known from previous observations [6].

The strengths of our study include the quantification of the biomarkers in the most common neurodegenerative dementias relevant in the CJD differential diagnostic context, especially the rapidly progressing forms of AD. In addition, the availability of clinically well-characterized samples allowed investigating the influence of the time of blood collection in the biomarker concentrations, which is uncommon in this type of studies.

## Conclusions

In total, our data validate previous observations and show that plasma Nfl and t-tau are clinically relevant biomarkers for the diagnosis of CJD with the potential to become the first blood-based diagnostic biomarkers to be implemented in the clinical settings. Our data shows that CJD subtype and certain diagnoses in the control group are associated with different diagnostic accuracies of plasma Nfl and t-Tau, underlining the importance of a thorough consideration of differential diagnoses and suggesting potential benefit from a combined use of both biomarkers. Plasma t-tau may also contribute to predict disease duration in CJD, with implications in counseling and interpretation of clinical trials, whereas plasma Nfl is associated with disease stage and progression in AD.

## Supplementary Information

**Additional file 1. **Plasma Nfl and t-tau correlations in the study population. **A.** Correlation analysis between plasma Nfl and t-tau concentrations in the study population stratified by disease group. Correlation coefficients with 95% CI and associated *p* values derived from Spearman test analysis are indicated for each comparison. Statistically significant differences are shown in bold. **B.** Scatter plot with plasma Nfl and t-tau concentrations in CJD. HC: healthy controls, NND: neurological diseases without dementia, NND-Dem: neurological diseases with dementia, AD: Alzheimer’s disease, CJD: Creutzfeldt-Jakob disease, DLB/PDD: dementia with Lewy bodies/Parkinson’s disease dementia, FTD: fronto-temporal dementia and VaD: vascular dementia, cc: correlation coefficient, 95% CI: 95% confidence interval. **C.** Correlation analysis in CJD cases between plasma Nfl and plasma t-tau concentrations with CSF Nfl, CSF t-tau, CSF Aβ42, plasma YKL-40, plasma t-PrP concentrations. Correlation coefficients with 95% CI and associated *p* values derived from Spearman test analysis are indicated for each comparison. Association between plasma NFl and t-tau with CSF 14-3-3 positivity was analyzed using the Mann-Whitney U test. Number of paired cases used in the analysis is indicated. Statistically significant differences are shown in bold. **D.** Scatter plots with plasma Nfl vs. CSF Nfl and **E.** plasma t-tau vs. CSF t-tau concentrations in CJD. cc: correlation coefficient, 95% CI: 95% confidence interval, n: number. Nfl: neurofilament light, t-tau: total-tau, Aβ42: amyloid beta 42.

**Additional file 2 **Relationship between plasma t-tau and disease duration in CJD. **A.** Resultant hazard ratios (HR) and associated *p*-values from Cox PH models, in which disease duration was the response variable and the biomarker (in pg/mL) was the predictor, performed as explained in Statistical analysis. **B.** When allowing for non-linear relationships (using the multivariable fractional polynomial method), the best fit considering disease duration from disease onset was obtained with the logarithmic transformation of the biomarker data. The Cox PH model rendered a concordance of 0.6098 (SE = 0.0301).**C.** When disease duration was measured from blood uptake, the best fit model was obtained without the logarithmic transformation, and the resultant Cox PH model offered a concordance of 0.6080 (SE = 0.0359).

## Data Availability

The datasets used and analyzed during the current study are available from the corresponding author on reasonable request.

## Abbreviations

Aβ42: Amyloid β
AD: Alzheimer’s disease
AUC: Areas under the curve
CJD: Creutzfeldt-Jakob disease
CNS: Central nervous system
CSF: Cerebrospinal fluid
DLB: Dementia with Lewy bodies
FTD: Fronto-temporal dementia
HC: Healthy controls
MMSE: Mini Mental Status Examination
Nfl: Neurofilament light
NND: Non-neurodegenerative neurological disease without dementia controls
NND-Dem: Non-neurodegenerative neurological disease with dementia controls
PDD: Parkinson’s disease dementia
PH: Proportional hazards
p-tau: Phosphorylated-tau
rpAD: Rapid progressive AD
RT-QuIC: Real-time quacking induced conversion assay
ROC: Receiver operating characteristic
spAD: Slow progressive AD
t-tau: Total-tau
VaD: Vascular dementia
