# Supplementary material for: Diagnostic and prognostic value of plasma neurofilament light and total-tau in sporadic Creutzfeldt-Jakob disease
Source: Alzheimers Res Ther. 2021 Apr 21;13:86. doi: 10.1186/s13195-021-00815-6 (PMC8059191; doi:10.1186/s13195-021-00815-6)
Supplement: Supplementary file 1 — Additional file 1. Plasma Nfl and t-tau correlations in the study population. A. Correlation analysis between plasma Nfl and t-tau concentrations in the study population stratified by disease group. Correlation coefficients with 95% CI and associated p values derived from Spearman test analysis are indicated for each comparison. Statistically significant differences are shown in bold. B. Scatter plot with plasma Nfl and t-tau concentrations in CJD. HC: healthy controls, NND: neurological diseases without dementia, NND-Dem: neurological diseases with dementia, AD: Alzheimer’s disease, CJD: Creutzfeldt-Jakob disease, DLB/PDD: dementia with Lewy bodies/Parkinson’s disease dementia, FTD: fronto-temporal dementia and VaD: vascular dementia, cc: correlation coefficient, 95% CI: 95% confidence interval. C. Correlation analysis in CJD cases between plasma Nfl and plasma t-tau concentrations with CSF Nfl, CSF t-tau, CSF Aβ42, plasma YKL-40, plasma t-PrP concentrations. Correlation coefficients with 95% CI and associated p values derived from Spearman test analysis are indicated for each comparison. Association between plasma NFl and t-tau with CSF 14-3-3 positivity was analyzed using the Mann-Whitney U test. Number of paired cases used in the analysis is indicated. Statistically significant differences are shown in bold. D. Scatter plots with plasma Nfl vs. CSF Nfl and E. plasma t-tau vs. CSF t-tau concentrations in CJD. cc: correlation coefficient, 95% CI: 95% confidence interval, n: number. Nfl: neurofilament light, t-tau: total-tau, Aβ42: amyloid beta 42. [file 13195_2021_815_MOESM1_ESM.pdf]

Additional file 1

A

	cc	95% CI	p value
HC	-0.0588	-0.2963 to 0.1856	0.6288
NND	-0.1107	-0.5325 to 0.2432	0.4035
NND-Dem	0.6957	0.3090 to 0.8850	**0.0019**
AD	-0.1107	-0.4124 to 0.1757	0.3758
CJD	0.3676	0.1586 to 0.5450	**<0.001**
DLB/PDD	0.0908	-0.2650 to 0.4248	0.6098
FTD	0.2238	-0.4726 to 0.7481	0.5343
VaD	0.2027	-0.2638 to 0.5923	0.3783

B

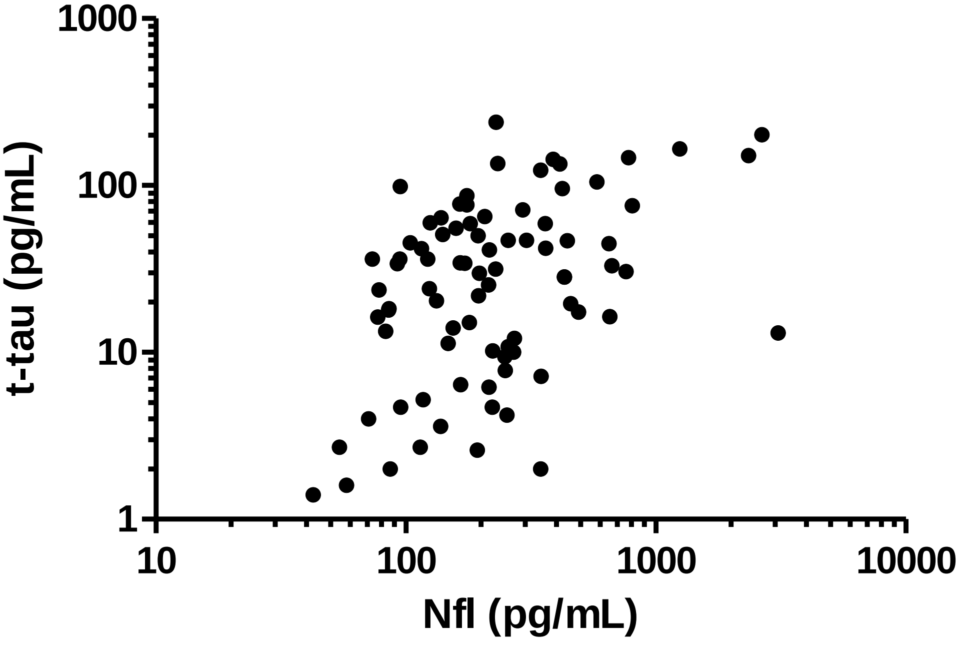

C

	Plasma Nfl			Plasma t-tau	
	n	cc (95% CI)	p value	cc (95% CI)	p value
CSF Nfl	51	0.5125 (0.2681-0.6949)	**0.0001**	0.1892 (-0.0995-0.4485)	0.1837
CSF t-tau	60	0.3266 (0.0716-0.5416)	**0.0109**	0.5425 (0.3278-0.7040)	**<0.001**
CSF 14-3-3	60	-	0.7954	-	**0.0211**
CSF Aβ42	40	0.1476 (-0.1811-0.4466)	0.3635	0.0677 (-0.2586-0.3916)	0.7618
Plasma YKL-40	42	0.4034 (0.1041-0.6357)	**0.0081**	0.0073 (-0.3058-0.3190)	0.9635
Plasma t-PrP	42	0.2609 (-0.0539-0.5316)	0.0926	-0.0785 (-0.3888-(-0.2479))	0.6304

Plasma Nfl and t-tau correlations in the study population and in CJD.

A. Correlation analysis between plasma Nfl and t-tau concentrations in the study population stratified by disease group. Correlation coefficients with 95% CI and associated p values derived from Spearman test analysis are indicated for each comparison. Statistically significant differences are shown in bold. **B**. Scatter plot with plasma Nfl and t-tau concentrations in CJD. HC: healthy controls, NND: neurological diseases without dementia, NND-Dem: neurological diseases with dementia, AD: Alzheimer’s disease, CJD: Creutzfeldt-Jakob disease, DLB/PDD: dementia with Lewy bodies/Parkinson’s disease dementia, FTD: fronto-temporal dementia and VaD: vascular dementia, cc: correlation coefficient, 95% CI: 95% confidence interval.

C. Correlation analysis in CJD cases between plasma Nfl and plasma t-tau concentrations with CSF Nfl, CSF t-tau, CSF Aβ42, plasma YKL-40, plasma t-PrP concentrations. Correlation coefficients with 95% CI and associated p values derived from Spearman test analysis are indicated for each comparison. Association between plasma NFl and t-tau with CSF 14-3-3 positivity was analyzed using the Mann-Whitney U test. Number of paired cases used in the analysis is indicated. Statistically significant differences are shown in bold. cc: correlation coefficient, 95% CI: 95% confidence interval, n: number. Nfl: neurofilament light, t-tau: total-tau, Aβ42: amyloid beta 42.
