# Supplementary material for: Diagnostic and prognostic value of plasma neurofilament light and total-tau in sporadic Creutzfeldt-Jakob disease
Source: Alzheimers Res Ther. 2021 Apr 21;13:86. doi: 10.1186/s13195-021-00815-6 (PMC8059191; doi:10.1186/s13195-021-00815-6)
Supplement: Supplementary file 2 — Additional file 2 Relationship between plasma t-tau and disease duration in CJD. A. Resultant hazard ratios (HR) and associated p-values from Cox PH models, in which disease duration was the response variable and the biomarker (in pg/mL) was the predictor, performed as explained in Statistical analysis. B. When allowing for non-linear relationships (using the multivariable fractional polynomial method), the best fit considering disease duration from disease onset was obtained with the logarithmic transformation of the biomarker data. The Cox PH model rendered a concordance of 0.6098 (SE = 0.0301).C. When disease duration was measured from blood uptake, the best fit model was obtained without the logarithmic transformation, and the resultant Cox PH model offered a concordance of 0.6080 (SE = 0.0359). [file 13195_2021_815_MOESM2_ESM.pdf]

A

	Disease duration from onset		Disease duration from blood uptake	
Predictor	HR	p value	HR	p value
Plasma t-tau (pg/mL)	1.004	0.037	1.005	0.022
Plasma Nfl (pg/mL)	1.000	0.700	1.000	0.981

B

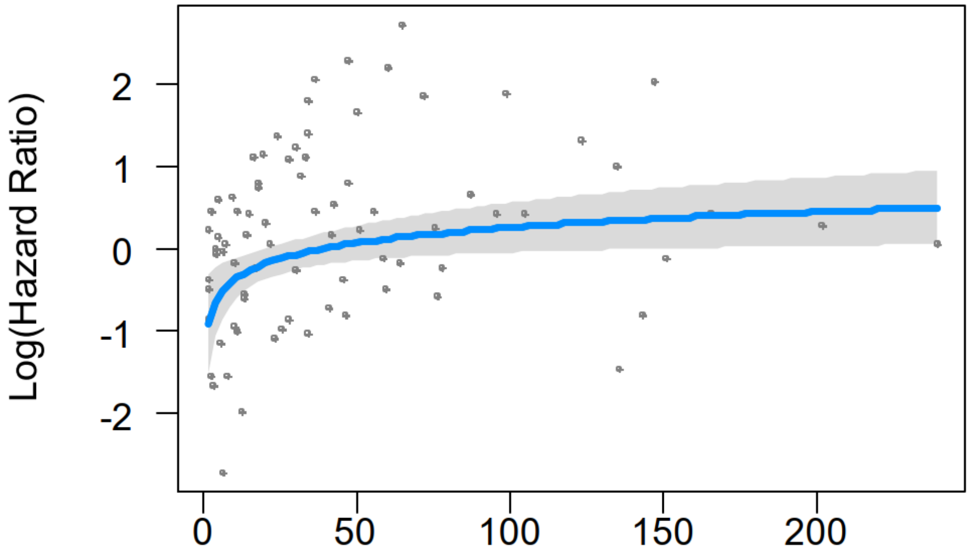

C

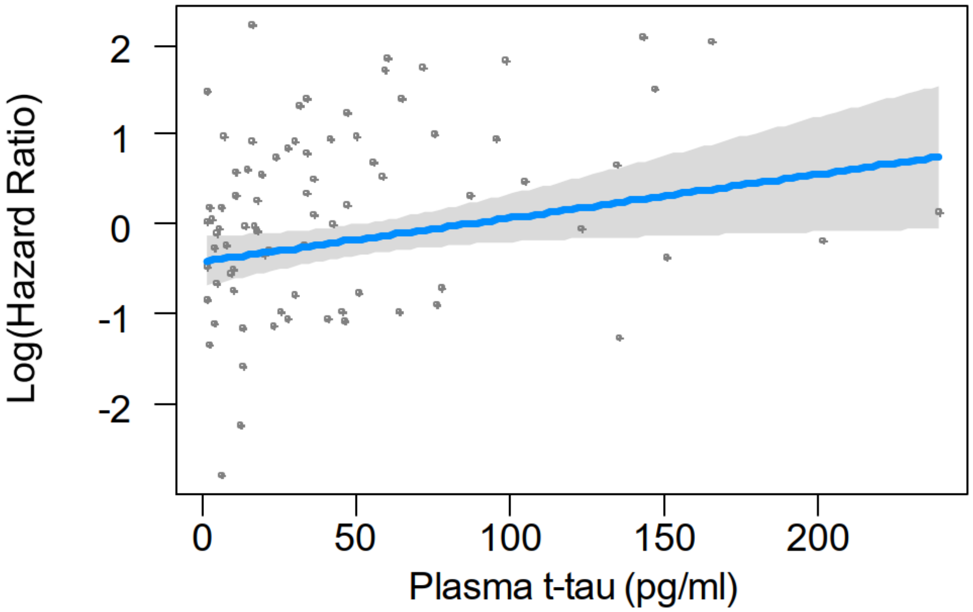

Relationship between plasma t-tau and disease duration in CJD

A. Resultant hazard ratios (HR) and associated p-values from Cox PH models, in which disease duration was the response variable and the biomarker (in pg/mL) was the predictor, performed as explained in Statistical analysis.

B. When allowing for non-linear relationships (using the multivariable fractional polynomial method), the best fit considering disease duration from disease onset was obtained with the logarithmic transformation of the biomarker data. The Cox PH model rendered a concordance of 0.6098 (SE=0.0301).

C. When disease duration was measured from blood uptake, the best fit model was obtained without the logarithmic transformation, and the resultant Cox PH model offered a concordance of 0.6080 (SE=0.0359).
